# Genome-wide identification of *GmEDS1* gene family members in soybean and expression analysis in response to biotic and abiotic stresses

**DOI:** 10.3389/fpls.2025.1554399

**Published:** 2025-04-29

**Authors:** Zhixian Liu, Jiahui Yang, Ziyu Yan, Lexiang Huang, Chengshun Xing, Miaoyu Zhao, Haiping Du, Milan He, Fanjiang Kong, Baohui Liu, Xiaohui Zhao

**Affiliations:** Guangdong Provincial Key Laboratory of Plant Adaptation and Molecular Design/Innovative Center of Molecular Genetics and Evolution, School of Life Sciences, Guangzhou University, Guangzhou, China

**Keywords:** soybean, *GmEDS1* family, expression pattern, biotic stresses, abiotic stresses

## Abstract

*Enhanced Disease Susceptibility 1* (*EDS1*), a key regulator in plant defense responses, plays central roles in resistance to stresses. Therefore, the identification and characterization of soybean *GmEDS1* family genes and verification of how these genes are associated with stresses are the focus of this study. We identified 11 *GmEDS1* genes, which all have lipase-like and EP (EDS1-PAD4-specific) conserved domains, they are unevenly distributed across six chromosomes, including tandem repetitions. Whole-genome duplication and segmental duplication events were the main reason for *GmEDS1* family expansion, and the family underwent purification selection during evolution. We detected 25 types of *cis*-regulatory elements, which enable *GmEDS1*s to respond to multiple signals. *GmEDS1*s are rapidly and strongly induced by drought, salt, the common cutworm, and soybean mosaic virus, indicating that they have important biological functions in coping with both abiotic and biological stresses. Furthermore, the expression levels of *GmEDS1*s differed between long-day and short-day conditions: it was very low under short-day conditions, which may increase the sensitivity of soybean to pathogens under short-day conditions. Overall, this study identified and characterized the members of the *GmEDS1* gene family in the soybean genome, and determined that *GmEDS1*s respond to both abiotic and biotic stresses, providing new key genes for soybean breeders.

## 1 Introduction

Soybean (*Glycine max* (L.) Merr.) is the most widely planted leguminous plant in the world, and it provides an important source of protein and oil for both humans and livestock. However, owing to the intensification of extreme weather and environmental degradation, soybean plants are negatively affected by many biotic and abiotic factors during growth and development. Drought severely affects the physiological activities of soybeans, including photosynthesis and nutrient transport, leading to a decrease in leaf area, limited accumulation of photosynthetic products, inadequate nutrition, and, ultimately, a decrease in yield (Li et al., 2023). And the similar phenotypes appear in plants subjected to salt stress. Soybean mosaic virus (SMV) disease, which is endemic throughout the world, causes mottling and necrosis of leaves, resulting in a large reduction in production (Li et al., 2010). Insect pest infestations are also an important factor that decreases soybean yield; among these pests, the common cutworm (CCW, *Spodoptera litura* Fabricius) is a widely distributed pest that feeds on over 300 crops and can cause 26% yield loss in the field (Saleem et al., 2016). Therefore, exploring the functions of stress resistance and stress tolerance genes will provide a powerful tool for improving and optimizing cultivated soybean varieties, while providing high-quality resources for soybean production and planting.

*Enhanced Disease Susceptibility 1* (*EDS1*) is an important defense gene, playing a crucial role in plant stresses. EDS1, Phytoalexin Deficient 4 (PAD4), and Senescence Associated Gene 101 (SAG101) are all related proteins in the EDS1 family, whose members are characterized by a lipase-like domain (LLD) at the N-terminus and a conserved EP (EDS1-PAD4-specific) domain at the C-terminus (Lapin et al., 2020). EDS1 can form heterodimers with PAD4 or SAG101, and can also form EDS1–PAD4–SAG101 triple complexes, linking the activation of pathogen immune receptors with the induction of host defense (Zhu et al., 2011; Lapin et al., 2020). EDS1–PAD4 heterodimers interact with Activated Disease Resistance 1 (ADR1) to mediate plant disease resistance and inhibit pathogen growth; An EDS1–SAG101 heterodimer can interact with N-Requirement Gene 1 (NRG1) to mediate plant cell death, leading to rapid tissue necrosis at sites of plant infection (Lapin et al., 2019; Dongus et al., 2022).

Pattern-triggered immunity (PTI) and effector-triggered immunity (ETI) are complex immune systems that have evolved in plants to respond to pathogen attacks. Plant resistance (R) proteins detect specific effector proteins secreted by pathogens, thereby activating ETI (Peng et al., 2018). Most R proteins contain a central nucleotide binding site (NBS) and leucine-rich repeats (LRRs), allowing R proteins to be further divided into two families based on the N-terminal sequence: the coiled-coil (CC)-NB-LRR family and the toll/interleukin 1 receptor like (TIR)-NB-LRR family (Wu et al., 2014). EDS1 can interact with TIR-NBS-LRR proteins in dicotyledonous plants (dicots), providing resistance to biological stress (Liu et al., 2002; Hu et al., 2005; Gao et al., 2010). In addition to participating in ETI, EDS1 and PAD4, as well as members of the ADR1 family, are also involved in PTI. PTI is triggered by interaction of an LRR receptor kinase and an LRR receptor protein RLP23 on the plasma membrane, forming a convergence point for plant defense signaling cascades, as EDS1–SAG101–NRG1 and EDS1–PAD4–ADR1 work synergistically in immunity (Dongus et al., 2022).

*PAD4* and *EDS1* help Arabidopsis (*Arabidopsis thaliana*) survive drought stress, regulating plant nutritional and reproductive growth (Szechyńska-Hebda et al., 2016). In grape (*Vitis vinifera*), *VvEDS1* increases resistance to powdery mildew caused by *Erysiphe necator*, an obligate biotrophic fungus, and pathogen infection induces upregulation of *VvEDS1* expression (Gao et al., 2014). Silencing *GmEDS1a*, *GmEDS1b*, and *GmPAD4* makes soybean more susceptible to infection by *Phytophthora sojae* and SMV, resulting in more severe infection symptoms or death, and exhibition of defect in SA accumulation (Wang et al., 2014), indicating that *GmEDS1* and *GmPAD4* may contribute to the defense response of soybean. For monocotyledonous plants (monocots), overexpression of wheat (*Triticum aestivum*) *TaEDS1* in susceptible wheat varieties leads to reduced growth of *Blumeria graminis* f. sp. *EDS1* regulates stress responses by participating in hydrogen peroxide/superoxide signaling (Chen et al., 2018). Under hypoxic conditions, Arabidopsis EDS1, PAD4, and LSD1 (LESION SIMULATING DISEASE 1) in hypocotyls jointly regulate the formation of lysogenic aerenchyma, which has enlarged air spaces that improve gas diffusion, in a process that involves the H_2_O_2_ and ethylene signaling pathways (Mühlenbock et al., 2007). Rice (*Oryza sativa*) *OsEDS1* enhances the heat stress resistance of rice plants by stimulating catalase activity and promoting the clearance of H_2_O_2_ (Liao et al., 2023).

In this study, we identified and comprehensively analyzed the members of the *GmEDS1* gene family in soybean, characterizing basic parameters of *GmEDS1* family proteins, gene structure and expression, and chromosomal locations. We also conducted analyses of phylogenetic relationships, *cis*-acting elements, and interaction protein networks. Expression analysis showed that *GmEDS1* genes were rapidly induced by a variety of biotic and abiotic stresses, including salt, drought, CCW infestation, and soybean mosaic virus infection. Thus, our findings lay a foundation for further elucidation of the critical stress response mechanisms regulated by *GmEDS1*, while providing a new perspective and tools for breeding for stress resistance in soybean.

## 2 Materials and methods

### 2.1 Plant materials and growth conditions

For this study, soybean variety Williams 82 (Wm82) seeds were planted in the greenhouse maintained at 25°C and 60% humidity, and with an average photon flux of 250 µmol m^−2^ s^−1^. Plants were grown with either long-day (LD) conditions of 16 hours of light and 8 hours of darkness or short-day (SD) conditions of 12 hours of light and 12 hours of darkness.

Soybean mosaic virus (SMV) strains SC3 and SC7, preserved by the Innovative Center of Molecular Genetics and Evolution Laboratory of Guangzhou University, were used and cultured in the SMV-susceptible soybean germplasm Nannong 1138-2 (NN1138-2).

Common cutworm (CCW, *Spodoptera litura* Fabricius) larvae were raised on an artificial diet at 25°C, under LD conditions.

### 2.2 Identification and annotation of *GmEDS1* genes

After downloading the soybean GFF file and the AtEDS1 family protein sequences from the Phytozome database (https://phytozome-next.jgi.doe.gov/), we predicted the conserved domains of AtEDS1 family proteins using the Pfam database online website (https://www.ebi.ac.uk/interpro/entry/pfam/) (Blum et al., 2025). We downloaded the hidden Markov model (HMM) profile from the InterPro website (https://www.ebi.ac.uk/interpro/download/Pfam/) (Blum et al., 2025). Candidate proteins were chosen as those containing at least these two domains: the lipase 3 domain (Pfam: PF01764) and the EP domain (Pfam: PF18117) (Lapin et al., 2019). We then used TBtools to convert the soybean genome annotation file into a protein sequence file, followed by BLASTp analysis to compare the AtEDS1 sequence with soybean protein sequences to identify all soybean proteins with similar sequences (Chen et al., 2023). Finally, we used the HMM method to identify soybean proteins containing both the lipase 3 and EP domains, and searched for corresponding gene annotations in the Phytozome database to screen for all members of the GmEDS1 family.

### 2.3 Prediction of physicochemical properties of soybean GmEDS1 proteins

To determine the numbers of amino acid residues, molecular weights, isoelectric points, and average hydrophobicity indexes of the GmEDS1 proteins, a detailed physicochemical analysis of members of the GmEDS1 protein gene family was conducted using Prot Param in the online software ExPASy (https://web.expasy.org/protparam/). Subcellular localizations of proteins were predicted using WoLF PSORT (https://psort.hgc.jp/).

### 2.4 Phylogenetic analysis of EDS1 proteins

Using the amino acid sequences of soybean GmEDS1s and their orthologs in Arabidopsis, alfalfa (*Medicago sativa*), tomato (*Solanum lycopersicum*), rice, maize (*Zea mays*), and sorghum (*Sorghum bicolor*) that we obtained from the Phytozome database, a multiple alignment was constructed using the neighbor-joining (NJ) method of MEGA 11 (Tamura et al., 2021), with the bootstrap replication set to 1000. The phylogenetic tree was beautified by ChiPlot (https://www.chiplot.online/) (Xie et al., 2023).

### 2.5 Gene structure and conserved motifs of *GmEDS1* genes

The positions and numbers of exons and introns of each *GmEDS1* gene were determined using TBtools software, based on the soybean GFF file downloaded from the Phytozome database. The GmEDS1s protein sequences were extracted, and the conserved motifs were predicted and analyzed using the MEME website (https://meme-suite.org/meme/). The maximum number of motifs was set to 15, with other parameters set to their default values. We used the CD search function at NCBI (https://www.ncbi.nlm.nih.gov/) to predict the structural domains of GmEDS1 proteins. Visualized them using TBtools (Chen et al., 2023), and used the Multiple Alignment tool of DNAMAN software for sequence alignment.

### 2.6 Chromosomal localization and synteny analysis

The chromosomal location of each *GmEDS1* gene was determined by using TBtools to download the GFF file of the soybean genome, and the ID list of GmEDS1 family members, from the Phytozome website data (Chen et al., 2023). Collinearity analysis of the *GmEDS1* genes was performed using the one-step MCScanX plug-in of TBtools and was visualized using the Advanced Circos plug-in (Chen et al., 2023). The Ka/Ks calculator in TBtools was used to calculate nonsynonymous substitution rates and synonymous replacement rates (Chen et al., 2023).

### 2.7 Analysis of *cis*−acting regulatory elements

We downloaded the promoter sequences (approximately 2 kb upstream of the transcription start sites) of soybean *GmEDS1* family genes from Phytozome, then used the PlantCARE online website (http://bioinformatics.psbugent.be/webtools/plantcare/html/) to predict and analyze promoter elements in these genes, remove the basic core elements (TATA-box and CAAT-box) of the promoter, and visualize them on TBtools (Chen et al., 2023).

### 2.8 Protein interaction network analysis

We searched the NCBI database for protein ID corresponding to *GmEDS1* family genes, and then used the STRING website (https://cn.string-db.org/) to perform protein–protein interaction analysis, adjusting the minimum required interaction score to high confidence (0.700). After obtaining the prediction results, we used the UniProt website (https://www.uniprot.org/) to find the gene number of the predicted interacting protein and search for the gene annotation at NCBI and Phytozome.

### 2.9 Spatial expression patterns of *GmEDS1* genes

To investigate the tissue expression patterns of *GmEDS1s*, samples were collected from Wm82 roots, cotyledons, and the shoot apical meristem tissues (SAM-V1) at the stage of full extension of true leaf. The first trifoliate leaves, petioles and stems were taken when the first trifoliate leaf was fully expanded. Flower buds, open flowers and pods were taken when they set. Three independent biological replicates were performed, the samples were immediately frozen in liquid nitrogen and stored at -80°C for RNA isolation. The soybeans were planted under LD conditions.

### 2.10 Abiotic and biotic stress treatments

Different photoperiod treatments: To investigate the expression of *GmEDS1s* under different photoperiod conditions, Wm82 seeds were planted in the soil under LD and SD conditions, respectively. Leaves were taken 20 days after emergence, and collected every 4 hours for a total of 24 hours. Three independent biological replicates were performed, the samples were immediately frozen in liquid nitrogen and stored at -80°C for RNA isolation.

Salt stress: Wm82 seeds were germinated in vermiculite for 5 days, after which they were transferred to a hydroponic device containing 1/2 × Hoagland nutrient solution (Coolaber, Beijing, China) under LD conditions in the greenhouse for 1 week. The one-week-old seedlings were treated with 1/2 × Hoagland nutrient solution containing 0 mM or 200 mM NaCl after 1 hour of light. Samples of the first trifoliate leaf samples were taken every 3 hours for a total of 48 hours. Three independent biological replicates were performed, the samples were immediately frozen in liquid nitrogen and stored at -80°C for RNA isolation.

Drought stress: Wm82 seedlings were placed in an equivalent volume of vermiculite under LD conditions for 2 weeks, and then subjected to drought conditions by withholding watering, with daily watered plants serving as a control group. The first trifoliate leaves were sampled on days 0 to 9 following initiation of the drought treatment. Three independent biological replicates were performed, the samples were immediately frozen in liquid nitrogen and stored at -80°C for RNA isolation.

CCW infestation: After Wm82 plants were cultured in the greenhouse under LD conditions for 2 weeks, two third-instar CCW larvae were placed on seedling leaves and fixed in position on the leaves with white mesh bags. Leaf samples were taken at 0, 4, 8, 12, 16, 20, and 24 hours after treatment. Three independent biological replicates were performed, the samples were immediately frozen in liquid nitrogen and stored at -80°C for RNA isolation.

SMV infection: When Wm82 grew to the true leaf stage under LD conditions, virus strains SC3 and SC7 were inoculated onto leaves using the following procedure. Leaves exhibiting an obvious SMV phenotype, mosaic, curling, wrinkled, and necrosis, on susceptible NN1138-2 plants were sampled. Leaves were then placed in 0.01 M sodium dihydrogen phosphate buffer (3-5 mL/g leaf tissue, pH 7.4), 1 g of quartz powder was added, and the mixture was ground with a mortar and pestle and rubbed onto the true leaves of one-week-old Wm82 seedlings with a brush. Virus-inoculated leaves were rinsed with tap water after inoculation. Wm82 leaves were sampled at 0, 2, 4, 6, and 8 days post inoculation (dpi). Three independent biological replicates were used, the samples were immediately frozen in liquid nitrogen and stored at -80°C for RNA isolation.

### 2.11 RNA extraction and RT-qPCR analyses

Total RNA was extracted using the RNA Extraction Kit (CWBIO, Jiangsu, China). This total RNA was reverse-transcribed to produce first-strand cDNA using the HiScript III RT SuperMix for qPCR (+gDNA wiper) (Vazyme, Beijing, China). The quantitative real-time PCR reactions were performed on a real-time Roche PCR instrument using the ChamQ SYBR qPCR Master Mix (Vazyme, Beijing, China). The internal reference gene was soybean *β-Tubulin*, and the relative expression level of the target gene was calculated using the $2^{-\text{ΔΔCT}}$ method. In the expression profile analysis, significance analysis was carried out using the Student *t*-test, indicate the level of significance with asterisks (*P< 0.05, **P< 0.01, and *** *P*< 0.001). Sequences of primers used for this study are provided in **Supplementary Table S1**.

## 3 Results

### 3.1 Identification and physicochemical analysis of soybean *GmEDS1* gene family members

To identify the GmEDS1 family numbers in soybean, we performed BLASTp analysis on the soybean genome by querying it with the reported sequence of AtEDS1 family proteins (Wagner et al., 2013). Through conservative domain analysis, a total of 11 candidate GmEDS1 family members, each containing a lipase 3 domain and an EP domain, were identified.

To gain a clearer understanding of the functions of soybean GmEDS1s, we analyzed the following physicochemical properties of each of these predicted GmEDS1 proteins: its chromosome (Chr) location, position coordinates, open reading frame length, amino acid (aa) number, molecular weight, theoretical pI (isoelectric point), and predicted subcellular localization (**Supplementary Table S2**). The 11 GmEDS1 family protein sequences were between 523 and 633 aa in length, with molecular weights ranging from 58.34 to 72.02 kDa. The range of pI values was 6.01–8.31, with six genes having a pI of greater than 7, indicating that GmEDS1 protein is typically alkaline and positively charged at neutral pH. The aliphatic index (AI) of GmEDS1s ranged from 74.76 to 86.06. The instability coefficients of GmEDS1s were 39.11–54.72. Generally, if this coefficient is less than 40, the protein is classified as a stable protein, and a value greater than 40 indicates an unstable protein. Most GmEDS1s were predicted to be unstable, because 90.9% of the instability coefficients were greater than 40. The grand average of hydropathicity (GRAVY) is defined as the ratio of the sum of hydrophilicity values of all amino acids in a sequence to the total number of amino acid residues. A larger negative value indicates stronger hydrophilicity; a larger positive value indicates stronger hydrophobicity (Kyte and Doolittle, 1982). All GmEDS1s were predicted to be hydrophilic proteins, because their GRAVY values were less than 0. In addition, GmEDS1 family proteins were predicted to be localized in the nucleus, cytoplasm, and chloroplasts. The physicochemical properties among GmEDS1 family members are relatively similar, suggesting that they may have similar functional roles.

### 3.2 Phylogenetic comparison of EDS1 proteins from different species

**Figure 1:**
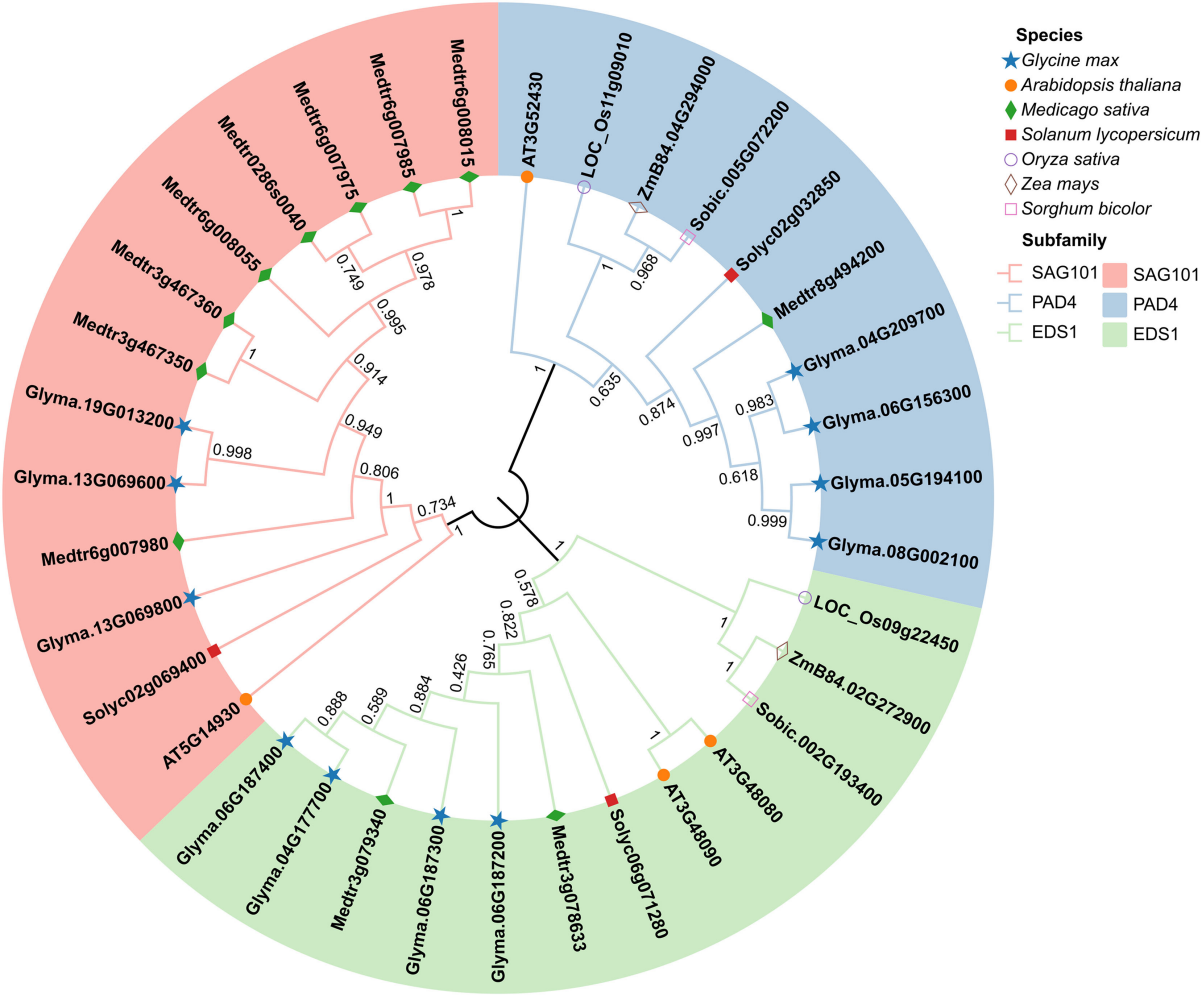
A phylogenetic tree of EDS1 family proteins from key dicots (solid symbols) and monocots (open symbols). The tree was constructed using protein sequences of EDS1 family members from *Glycine max* (blue stars), *Arabidopsis thaliana* (orange circles), *Medicago sativa* (green diamonds), *Solanum lycopersicum* (red squares), *Oryza sativa* (open purple circles), *Zea mays* (open brown diamonds), and *Sorghum bicolor* (open pink squares). Construction used the neighbor-joining (NJ) method with 1000 bootstrap replications in MEGA 11. The SAG101 (pink), PAD4 (blue), and EDS1 (green) subfamilies are distinguished by color.

To investigate the phylogenetic relationships of EDS1s in soybean and other species, we analyzed 35 EDS1 protein sequences from seven species. This includes soybean (11 sequences), model plant of dicots Arabidopsis (4), model plant of monocots rice (2), legume plant alfalfa (11), as well as some crops tomato (3), maize (2), and sorghum (2). Our phylogenetic analysis showed that all EDS1s were clustered into three distinct subfamilies: EDS1, PAD4, and SAG101 (**Figure 1**). This result is consistent with the classification of the EDS1s in Arabidopsis (Feys et al., 2005; Zhu et al., 2011). The members of the GmEDS1 family are almost evenly distributed across the three subfamilies (**Figure 1**). We thus named the 11 soybean *EDS1* family genes *GmEDS1a, b, c*, and *d*; *GmPAD4a, b, c*, and *d*; and *GmSAG101a, b*, and *c*. Soybean EDS1s were evenly distributed among the three subfamilies, whereas those of alfalfa were found primarily in the SAG101 subfamily (**Figure 1**). It is quite interesting to note that the monocots studied had no members in the SAG101 subfamily. This suggests that the functions of the subfamily members may have differentiated in monocots vs. dicots during evolution.

### 3.3 Chromosomal distribution and synteny of *GmEDS1* genes in soybean

**Figure 2:**
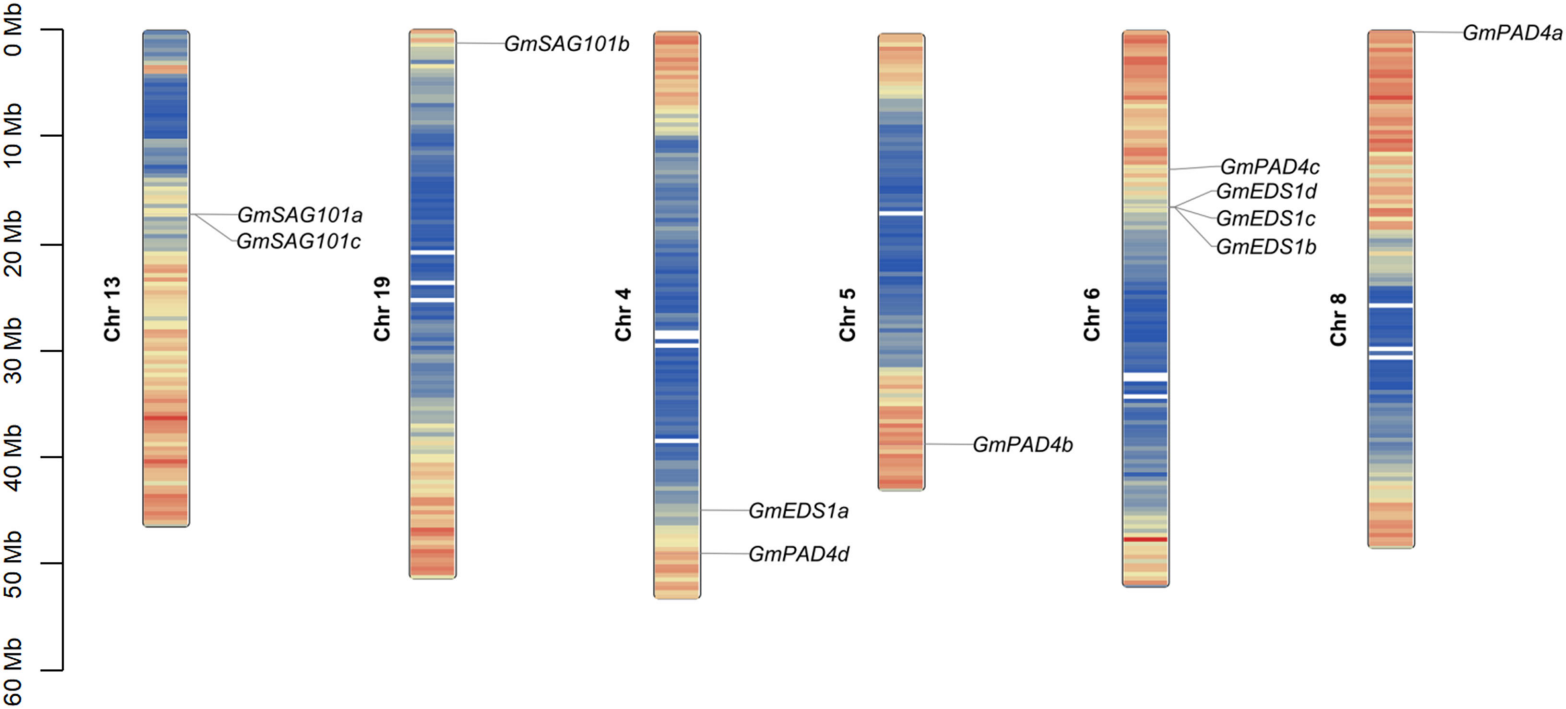
Chromosomal distribution and inter-chromosomal relationship of *GmEDS1* family genes. Each *GmEDS1* was mapped to its chromosomal position using its physical position in the soybean genome. The chromosome number is noted beside each chromosome. The scale bar at the left indicates the distance from the chromosome end in megabases (Mb). The color on the chromosome indicates the density of genes in that chromosome region, with red indicating the highest density and blue indicating the lowest density.

To analyze the phylogenetic relationships among *GmEDS1* family genes, their chromosomal position, collinearity, and evolution were determined. The physical locations of the 11 *GmEDS1* genes in the soybean genome are unevenly distributed in high-density gene regions of six of the chromosomes, with different numbers of *GmEDS1* genes on each chromosome (**Figure 2**). *GmPAD4* subfamily members are distributed among Chr4, Chr5, Chr6, and Chr8, with three located on the telomeric region of the chromosome and one relatively close to the centromere. One of the *GmSAG101* subfamily genes is at the end of Chr19, and two are in the middle of Chr13. *GmSAG101a* and *GmSAG101c* are very close together, with only one gene in between. Of the four soybean *GmEDS1* homologous genes, three of them are closely linked in the middle of Chr6.

**Figure 3:**
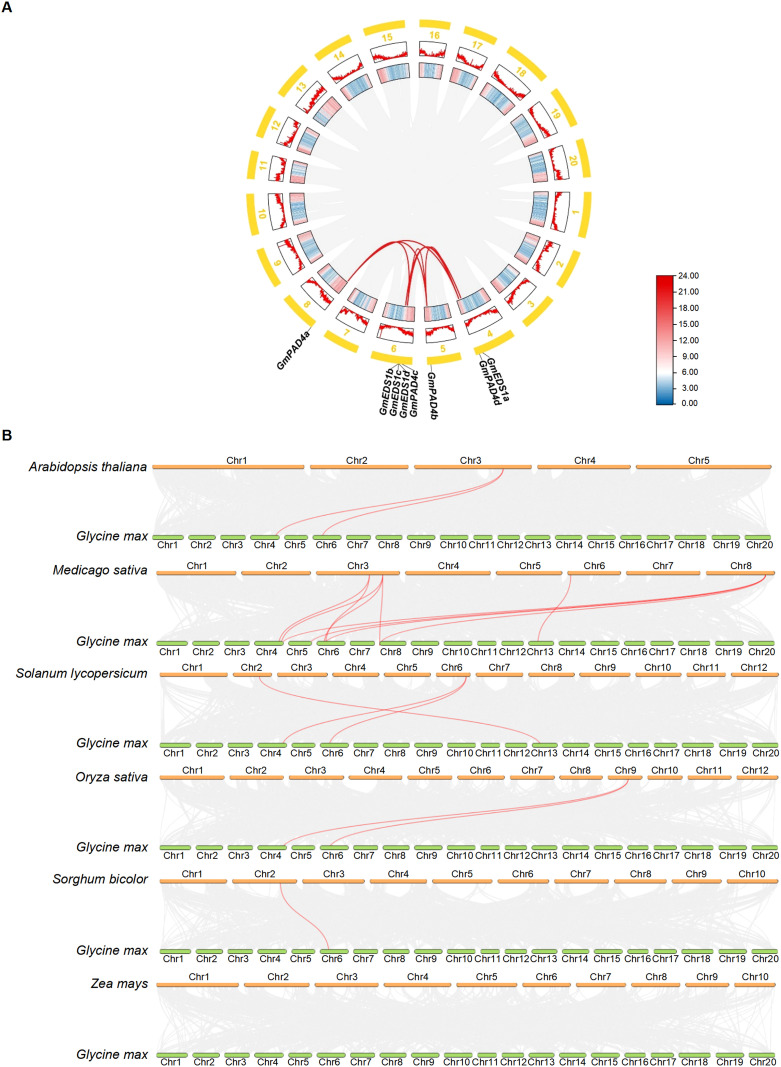
Synteny analysis of *EDS1* family genes in soybean and other key plants. **(A)** Synteny analysis of soybean *GmEDS1* genes. Red curved lines connect duplicated gene pairs. The outer circle illustrates the positions of these genes, with thick yellow lines corresponding to different chromosomes (Chr1–Chr20). Red zigzag lines in boxes below the yellow lines highlight the gene density on each chromosome, with higher densities being further from the center. The inner boxes of the diagram further emphasize gene density within the chromosomes, with red denoting the highest density and blue indicating the lowest density. **(B)** Synteny analysis comparing soybean *EDS1* gene locations with those of other key species. Lines in the background indicate collinear blocks between *Glycine max* and the indicated plant. Red lines connect syntenic *EDS1* gene pairs.

Intraspecific collinearity analysis of the soybean genome showed that there were seven pairs of fragment duplication events in the 11 *GmEDS1* genes (*GmEDS1a*/*GmEDS1d*, *GmPAD4a*/*GmPAD4b*, *GmPAD4a*/*GmPAD4c*, *GmPAD4a*/*GmPAD4d*, *GmPAD4b*/*GmPAD4d*, *GmPAD4b*/*GmPAD4c* and *GmPAD4c*/*GmPAD4d*) (**Figure 3A**). Among them, six pairs belong to the *GmPAD4* subfamily, and one pair belongs to the *GmEDS1* subfamily, indicating that the expansion of the *GmPAD4* family may have depended on segmental replication events. The sequence similarity among *GmPAD4* subfamily proteins is 73.96% (**Supplementary Figure S2**), which is consistent with this result. The Ka/Ks ratios of the seven pairs of fragment duplication events were all less than 1 (**Supplementary Table S3**), indicating that purifying selection, which can keep the gene stable and preserve its function, occurred in *GmPAD4* and *GmEDS1* during the evolutionary process.

To elucidate the evolutionary relationships among the members of the *EDS1* gene family, we constructed an interspecies collinearity map of seven species (**Figure 3B**) comparing soybean with Arabidopsis, alfalfa, tomato, rice, sorghum, and maize. The strength of association with *GmEDS1* genes, from highest to lowest, was alfalfa (10 pairs), tomato (3), Arabidopsis (2), rice (2), sorghum (1), and maize (0) (**Figure 3B**; **Supplementary Table S4**). There were 15 pairs of orthologs in dicots, and only 3 pairs in monocots. Notably, *GmEDS1d* is related to 5 orthologous genes, and *GmEDS1a* is related to 4 orthologs (**Figure 3B**; **Supplementary Table S4**). Notably, in monocots, all of the orthologous pairs, excluding *PAD4* and *SAG101*, belong to the *EDS1* subfamily. This suggests that the *EDS1* subfamily members play much more important roles, and the orthologous pairs may have existed before the split between dicots and monocots.

### 3.4 Conserved domain and phylogenetic analysis of the *GmEDS1* gene family

**Figure 4:**
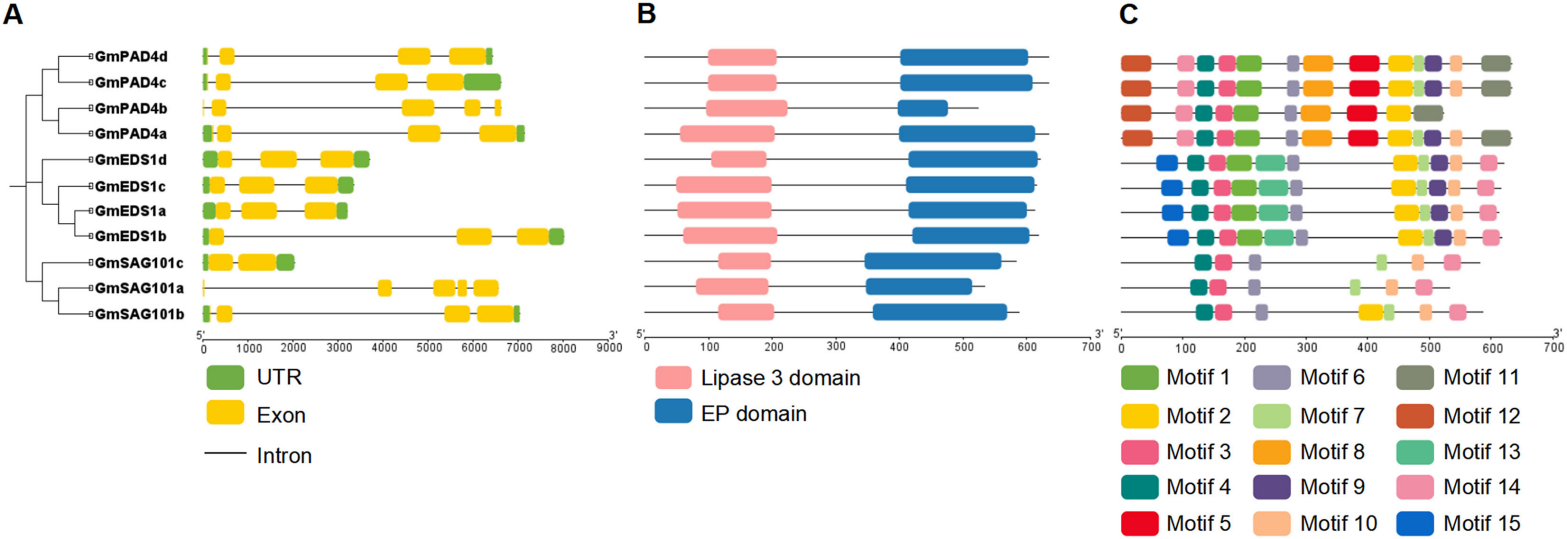
Structure of soybean *GmEDS1* family members. **(A)** Phylogenetic relationships among *GmEDS1* family members (left) and exon–intron structural diagrams of *GmEDS1* family genes (right). The green boxes represent 3′ and 5′ UTR regions, the yellow boxes represent exons, and the black connecting lines represent introns. The scale at the bottom represents gene length in base pairs. **(B)** Predicted conserved domains of GmEDS1 family proteins. The length of each line represents the protein length, the pink boxes are the lipase 3 domain, and the bule boxes are the EDS1 EP domain. The scale at the bottom represents protein length in amino acid residues. **(C)** Distribution of conserved motifs in GmEDS1 family proteins. The motif patterns highlight the similarities within subfamilies. The scale at the bottom represents protein length.

The gene structures and conserved domains of the *GmEDS1* gene family members were analyzed based on their coding sequences and gene annotation. The numbers of exons in the *GmEDS1* genes were similar, with most members containing three exons (**Figure 4A**). All GmEDS1 proteins contained both an EP domain and a lipase 3 domain (**Figures 4B**, **Supplementary Figures S1**-**S3**). There is a G-X-S-X-G motif and an S-D-H catalytic triad (**Supplementary Figures S1**-**S3**), characteristic of most catalytic α/β hydrolase proteins and relatively conserved in the GmEDS1 family (Brenner, 1988). Comparing the EDS1 protein sequences of soybean and Arabidopsis, it was found that the sequence similarity of EDS1, PAD4, and SAG101 proteins was 61.77%, 72.03%, and 58.62%, respectively, indicating that the PAD4 family is relatively conserved in evolution (**Supplementary Figures S1**-**S3**). Meanwhile, it can be observed that the S-D-H catalytic triad is completely conserved in the EDS1 and PAD4 families (**Supplementary Figures S1**-**S3**), but not conserved in the SAG101 family, which may affect the α/β hydrolase activity of SAG101. The activity of hydrolases is not essential for immune function, but it is necessary for EP domain stability and resistance (Wagner et al., 2013).

To further investigate the diversity of GmEDS1 family proteins, we performed a more comprehensive motif analysis and found that 15 motifs were predicted using the MEME Suite website (**Figures 4C**, **S4**). Among them, motifs 3, 4, 6, and 7 were shared by all GmEDS1 family members, motifs 5, 8, 9, 11, 12, and 14 were unique to the GmPAD4 subfamily, and motifs 13 and 15 were unique to the GmEDS1 subfamily; however, there was no motif unique to the GmSAG101 subfamily. The types and conservation of motifs within a subfamily are high, indicating that members with close phylogenetic relationships have proteins with similar biological functions. However, the types of motifs contained in different subfamilies are quite different, indicating that the functions of proteins among different subfamilies are also likely to differ.

### 3.5 Analysis of *cis*-regulating elements in the promoters of *GmEDS1* family genes

**Figure 5:**
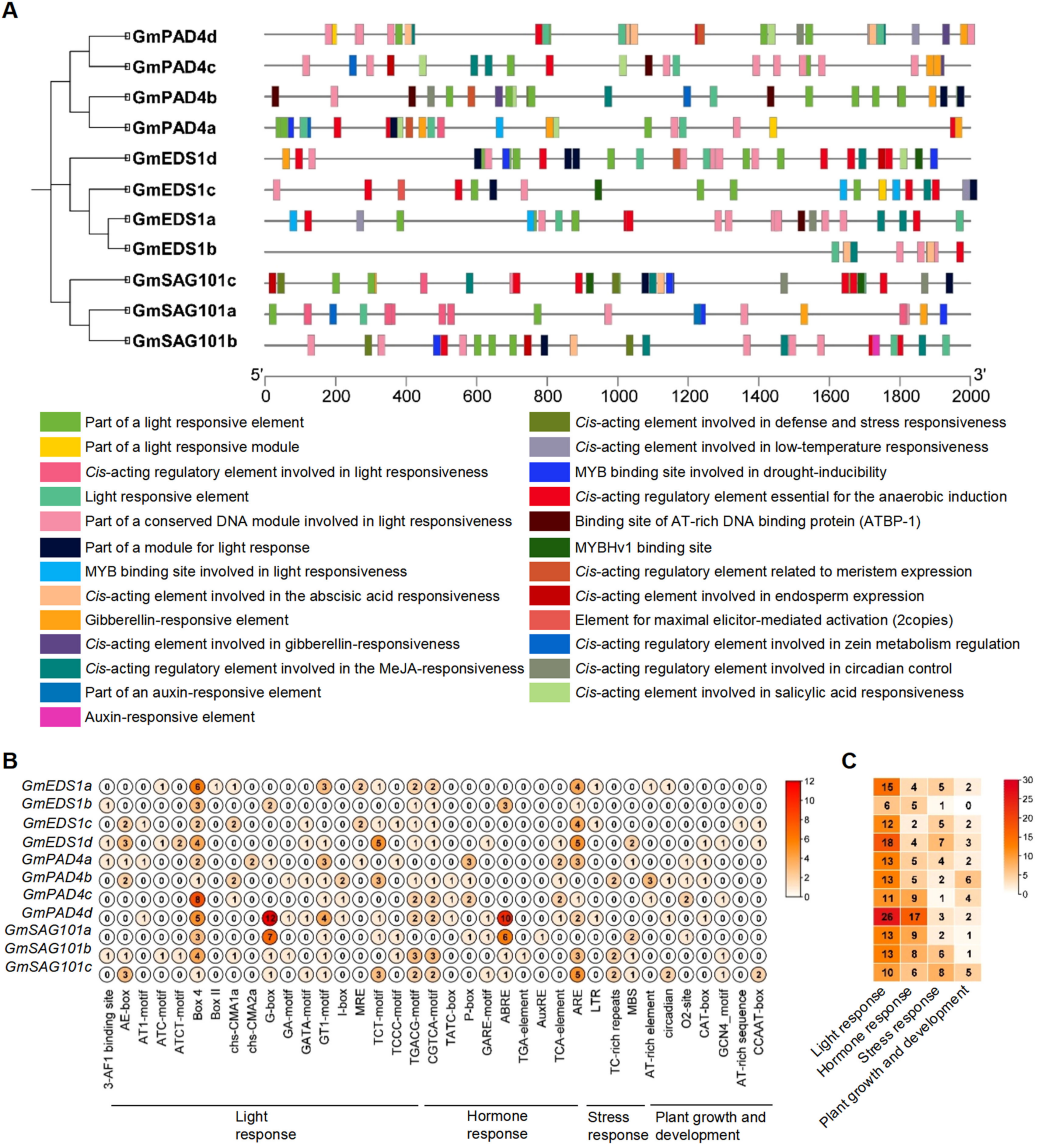
Analysis of *cis*-acting elements of *GmEDS1* family gene promoters. **(A)** The positions of the 25 *cis*-acting elements with different functions in the upstream 2000-bp promoter region of the *GmEDS1* family genes. Different colors indicate different *cis*-acting elements, with the scale indicating the length of the promoter region in bp. Below are the corresponding relationships between different colors and the functions of *cis*-acting components. **(B)** The number of each different type of *cis*-acting element in each *GmEDS1* family gene. The names and functional classifications of the *cis*-acting elements are shown at the bottom; the numbers inside the circles (0–12) indicate the number of elements, with darker colors representing higher numbers of elements. **(C)** The number of *cis*-acting elements involved in different biological process in the *GmEDS1* family. At the bottom is the functional classification of *cis*-acting elements. The numbers in the box represent the number of different types of elements, and the color key on the right shows the relationship between the number of elements and the color of the box.

Gene function is largely determined by the type and number of *cis* elements in the gene promoter. To explore the potential role of *GmEDS1* genes, 2,000 bp of sequences upstream of the transcription start site were analyzed. We found a total of 25 categories of *cis-*regulating elements (**Figure 5A**), based on their functions further classified them into 4 biological processes refining into 37 different types: light response (17 types), plant hormone regulation (9), stress response (4), and growth and development (7) (**Figures 5B, C**; **Supplementary Table S5**). Of them, light-responsive *cis-*elements were the most prevalent (**Figure 5C**), suggesting that *GmEDS1* family members are likely to be induced by specific light signals while eliciting defensive responses during soybean growth and development. In addition, a variety of hormone-responsive *cis-*acting regulatory elements were discovered, including elements involved in abscisic acid (ABA), methyl jasmonate (MeJA), salicylic acid (SA), gibberellic acid (GA), and auxin responses (**Figure 5A**; **Supplementary Table S5**). Moreover, *cis-*elements associated with defense, stress, drought, anaerobic conditions, and low temperature were also widely distributed in the promoter regions of the various soybean genes. It is worth noting that the G-box and ABA-responsive elements (ABRE) of *GmPAD4d* promoter were the most abundant (**Figures 5B, C**). The number and diversity of *cis*-regulating elements discovered in this analysis highlights how the *GmEDS1* gene family members are involved in a wide variety of responses to biotic and abiotic stresses. Therefore, the *GmEDS1* family is a key factor in soybean growth and development, and is important in helping plants cope with adversity.

### 3.6 GmEDS1 protein–protein interaction network analysis

**Figure 6:**
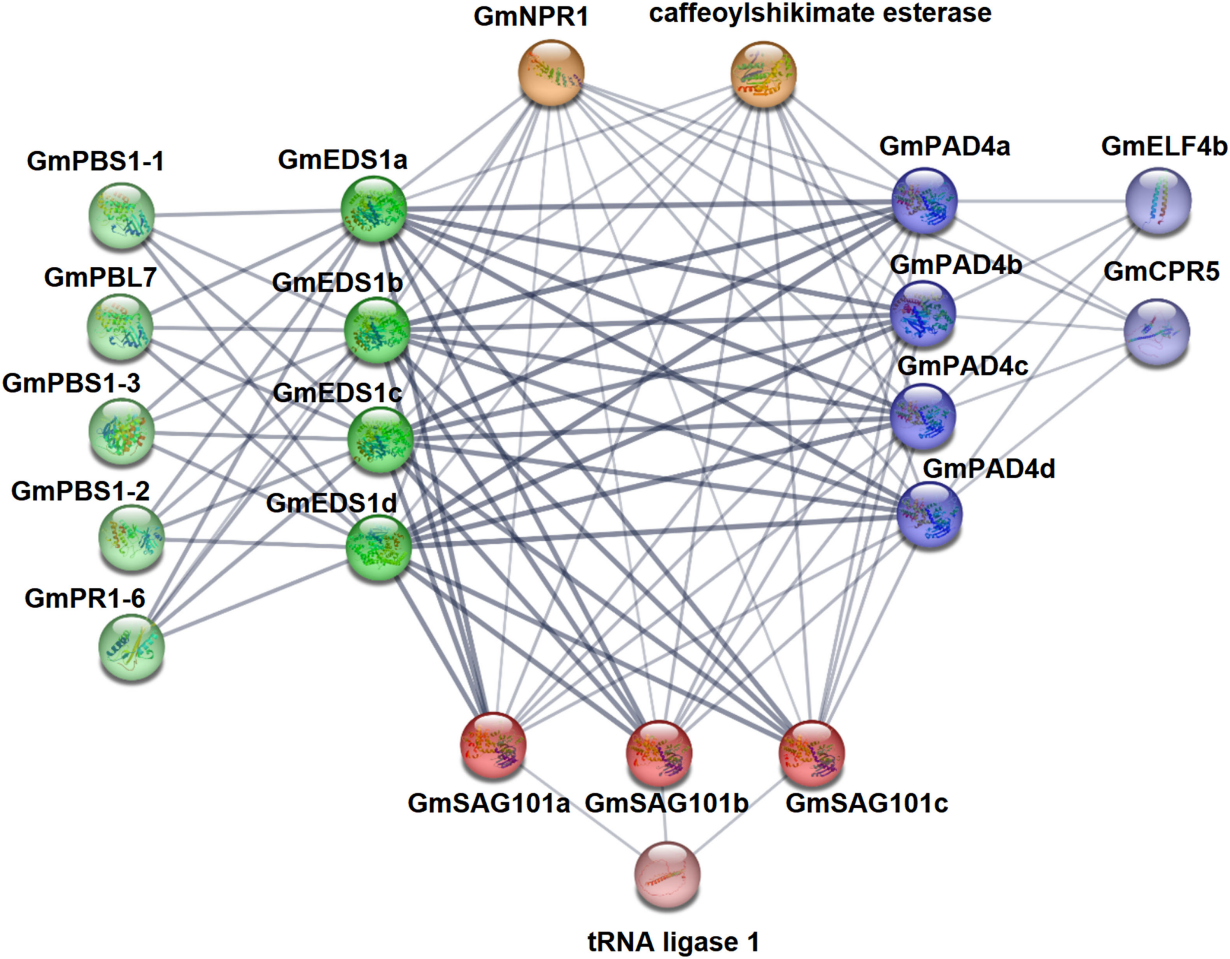
Protein interaction prediction network of GmEDS1 family proteins. Use STRING website to predict protein interaction network of GmEDS1 family members. The labeling around the protein is its name. The dark green balls represent the GmEDS1 subfamily proteins, the dark blue colors represent the GmPAD4 subfamily proteins, the dark red colors represent the GmSAG101 subfamily proteins, the light-colored balls near them represent predictive proteins that interact with them, and the orange balls represent predicted proteins that interact with all GmEDS1 family members. The thickness of the lines connecting each protein represents confidence: the thicker the line, the higher the interaction strength.

In the organism, proteins do not exist alone, but through protein–protein interactions they form complex networks that achieve proper biological functions and physiological activities. Therefore, we predicted the likely protein–protein interactions of the 11 soybean GmEDS1 proteins using the STRING database. We found a strong interaction between GmEDS1s and either GmPAD4s or GmSAG101s. There was also an interaction between GmPAD4s and GmSAG101s, but the strength was lower than that with GmEDS1s (**Figure 6**; **Supplementary Table S6**). It is suggested that GmEDS1 members may form protein complexes to regulate soybean defense responses.

Most of the predicted GmEDS1-interacting proteins were related to immune responses. Among them, NPR1 (NONEXPRESSOR OF PATHOGENESIS-RELATED GENES 1) was predicted to interact with GmEDS1 members (**Figure 6**; **Supplementary Table S6**). NPR1 plays an important role in plant biotic and abiotic stress responses and is indispensable in plant immune responses (Backer et al., 2019). Activation of SA- and JA-induced responses is necessary for plant defense, and NPR1 can mediate crosstalk involving SA and JA/ET (jasmonic acid/ethylene), it can regulate SA accumulation, and it can activate appropriate plant defense signals (Backer et al., 2019).

GmPAD4 subfamily proteins were predicted to interact with CPR5 (CONSTITUTING PATHOGEN RESPONSE 5) and ELF4 (EARLY FLOWERING 4) (**Figure 6**; **Supplementary Table S6**). CPR5 plays a role in plant pathogen defense response, programmed cell death, cell wall biosynthesis, seed production, and regulation of senescence. It can also enhance resistance of Arabidopsis plants to heat stress through the SA pathway (Clarke et al., 2004; Wang et al., 2012). The circadian clock gene *ELF4* plays an important role in the integration and regulation of flowering and circadian rhythms in Arabidopsis (Doyle et al., 2002). *CO* (*CONSTANS*), *FT* (*FLOWERING LOCUS T*), and *GI* (*GIGANTEA*) are key photoperiodic flowering genes in Arabidopsis (Putterill et al., 1995; Fowler et al., 1999; Kardailsky et al., 1999), and ELF4 was shown to alter flowering time by impeding GI binding to the promoter of *CO* (Kim et al., 2013). Overexpression of *GmELF4* in Arabidopsis downregulates the expression of *CO* and *FT*, leading to delayed flowering (Marcolino-Gomes et al., 2017). Because the GmPAD4 subfamily genes are predicted to interact with GmELF4, this strongly suggests that they are involved in regulation of photoperiodic flowering in soybean.

PBS1 (AvrPphB SUSCEPTIBLE 1), PR1 (PATHOGENESIS RELATED 1), and PBL7 (serine/threonine protein kinase PBL7) were predicted to interact with GmEDS1 subfamily proteins (**Figure 6**; **Supplementary Table S6**). PBL7 plays a role in response to cold stress (Gao et al., 2022; Panahi and Shahi, 2024). AvrPphB (*Pseudomonas* biphasic avirulence protein B) can proteolytically cleave PBS1, and this cleavage is necessary for activating RPS5 (RESISTANCE TO PSEUDOMONAS SYRINGAE 5), an R protein that mediates the hypersensitivity reaction (Shao et al., 2003). *OsPBL1* (*Oryza sativa Arabidopsis PBS1*-*like 1*) is a homolog of *AtPBS1*, and exogenous application of SA upregulates *OsPBL1*, indicating that PBS1 plays a role in SA-mediated defense signal transduction (Lee and Kim, 2015). AtEDS1 is also involved in the SA pathway (Caldwell and Michelmore, 2009), so the interaction between PBS1 and EDS1 subfamily proteins may help to modulate the SA-mediated signaling pathway. PR1 is also an important component of the SA pathway and is commonly used as a marker gene for plant defense mechanisms (Shin et al., 2014; Lee and Kim, 2015). The results of the protein–protein interaction network analysis indicate that GmEDS1 can interact with many immune response–related proteins. Therefore, it may play important roles both in plant immune responses and in photoperiod regulation, based on the presence of so many light-responsive elements in the *GmEDS1* gene promoters.

### 3.7 *GmEDS1* genes response to photoperiod

**Figure 7:**
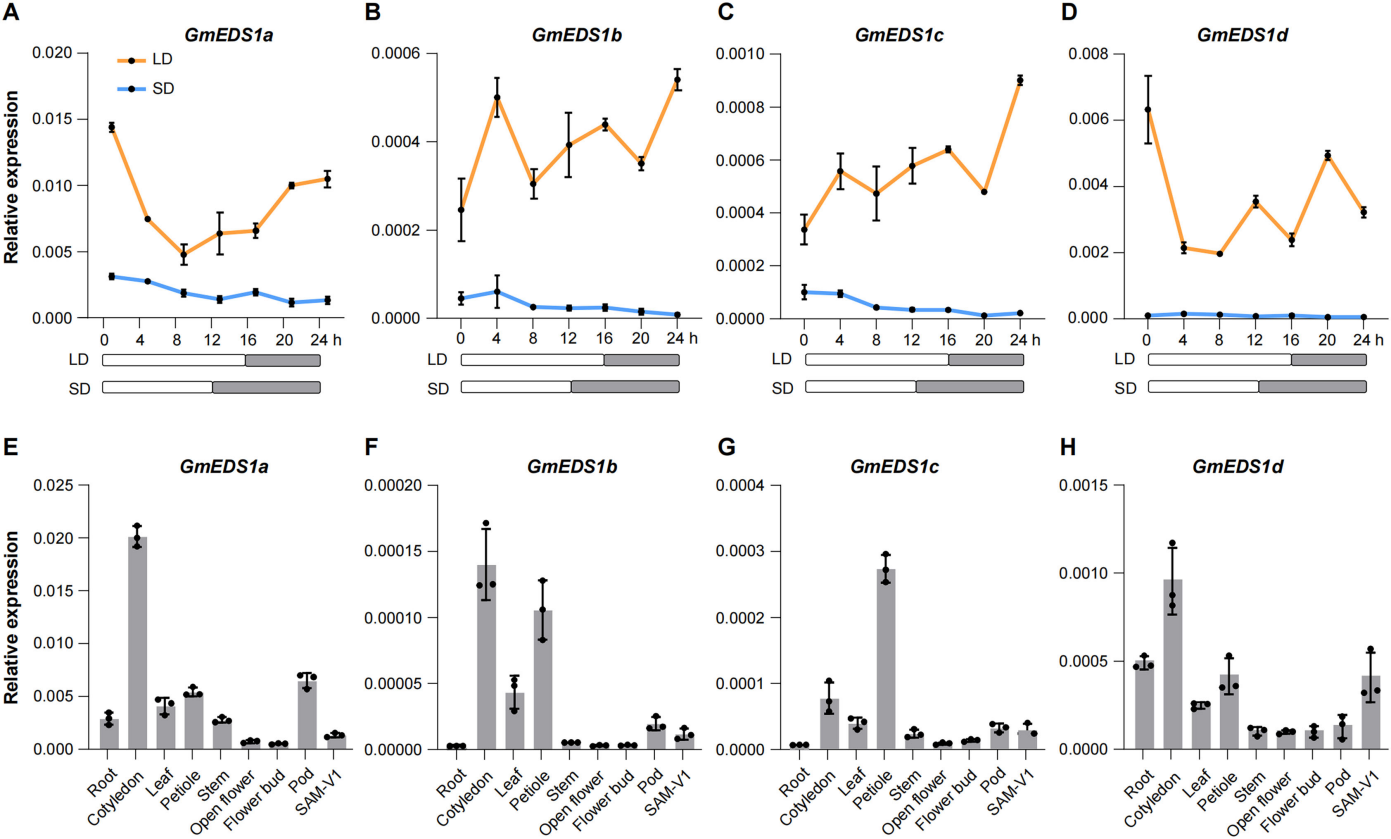
Expression patterns of *GmEDS1* genes. **(A–D)** Diurnal expression pattern of *GmEDS1* genes in leaves under LD (16-hour light/8-hour dark) and SD (12-hour light/12-hour dark) conditions. The rectangles below represent the light (white) and dark (gray) periods of the photoperiod, respectively. **(E–H)** Relative expression of *GmEDS1* genes in different tissues. Expression levels were normalized to that of *β*-*Tubulin*, which was used as a reference transcript. Data are means ± SD of three independent experiments.

Soybean is extremely sensitive to photoperiod, and because there were many light-responsive *cis-*elements in *GmEDS1* gene promoters, we monitored *EDS1* gene expression under different photoperiod conditions: LD (16-hour light/8-hour dark) conditions and SD (12-hour light/12-hour dark) condition. Expression levels of the four *GmEDS1* subfamily members were all much higher under LD conditions, indicating that *GmEDS1* expression was indeed influenced by photoperiod and that LD represents the induced condition (**Figures 7A–D**). Therefore, in the following research, we focused on the various physiological responses of soybean regulated by *GmEDS1* only under LD conditions.

To further understand the function of *GmEDS1* subfamily members, expression levels were measured in different tissues: root, cotyledon, leaf, petiole, stem, open flower, pod, and SAM-V1 tissues. Overall, *GmEDS1*s were expressed in all of the tissues tested, and were especially highly expressed in the cotyledon and petiole. The expression of *GmEDS1a* was higher than that of the other three *GmEDS1*s (**Figures 7E–H**). Based on its universal expression pattern in the various tissues, it is likely that *GmEDS1* members play a variety of important roles in plant growth and development.

### 3.8 *GmEDS1* genes respond to abiotic and biotic stresses

**Figure 8:**
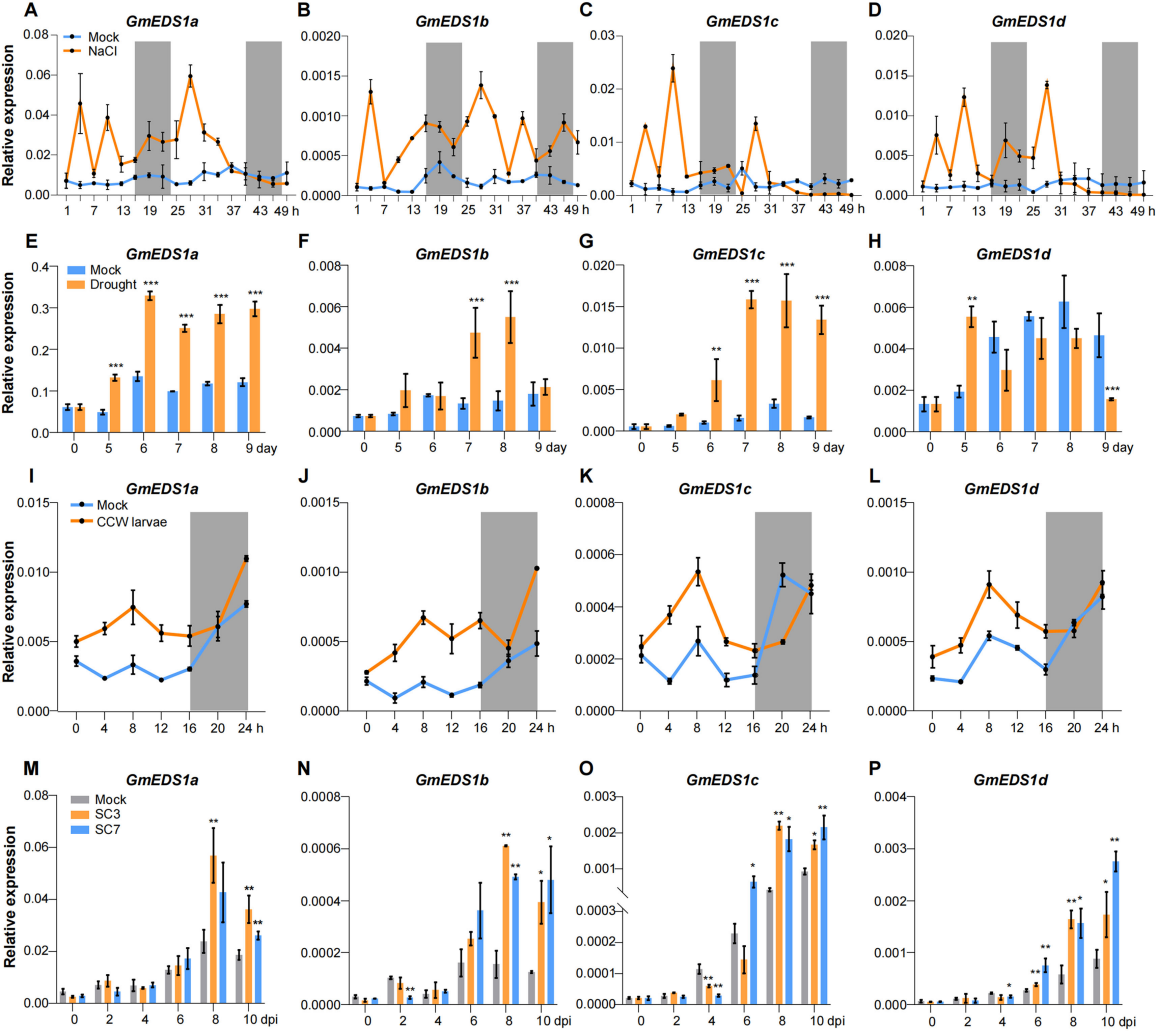
Expression analysis of *GmEDS1s* under abiotic and biotic stress. **(A–D)** Analysis *of GmEDS1s* expression for 48 hours following salt stress. Sampling began 1 hour after the light came on, with a 3-hour interval between sampling points. Soybean plants were treated with 0 mM NaCl (Mock, blue line) or 150 mM NaCl (orange line). The gray shading represents the dark phase of the photoperiod. **(E–H)** Analysis of *GmEDS1* expression levels during 9 days of drought treatment. Soybean plants were grown under control (blue line) or drought treatment by withholding watering (orange line) as described in Methods. **(I–L)** Expression levels of *GmEDS1*s in CCW larvae–infested plants within 24 hours after infestation. Samples were taken every 4 hours. Mock represents uninfected plants serving as a negative control. The gray shaded area represents the dark phase of the photoperiod. **(M–P)** Changes in expression levels of *GmEDS1*s within 10 days following SMV infection. The gray bars represent the expression levels of *GmEDS1*s in the absence of SMV infection; the orange and blue bars represent the expression levels of *GmEDS1*s following infection with SC3 or SC7, respectively. Relative expression levels were normalized to that of *β*-*Tubulin*, which was used as a reference transcript. Data are means ± SD from three biological replicates. Student’s *t* test. *P< 0.05, **P< 0.01 and ***P< 0.001, comparison of *GmEDS1*s transcription level between mock and treatment group.

Unlike animals, plants are sessile organisms that are unable to move away from any of the environmental challenges throughout their growth and development, inevitably facing both abiotic and biotic stresses. We measured *GmEDS1* gene expression in soybean plants under a variety of abiotic and biotic stress conditions such as salt, drought, SMV infection, and CCW infestation to determine how *GmEDS1* genes would respond to these stresses. In response to salt stress, all of the *GmEDS1* genes were rapidly and significantly upregulated within 3 hours, and the expression levels remained high for 30 hours (**Figures 8A–D**). After 5 days without watering, *GmEDS1a* and *GmEDS1d* were significantly induced; *GmEDS1c* and *GmEDS1b* showed significant increases at 6 and 7 days, respectively (**Figures 8E–H**). It is possible that each *GmEDS1* family member has a different degree of sensitivity to drought stress, which might explain why the response times were slightly different.

To test the effects of biotic stress on *GmEDS1* gene expression, we exposed plants to CCW larvae. In response to feeding, transcription levels of the *GmEDS1*s rapidly increased (**Figures 8I–L**). Notably, induction weakened or ceased during the night, probably because the larvae had stopped feeding during that time, reflecting how expression of the *GmEDS1* family genes is indeed aligned with stress responses. When soybean plants were infected with SMV, all of the *GmEDS1*s except *GmEDS1a* responded at 2 or 4 dpi (**Figures 8M–P**). With increased virus infection time, all of the *GmEDS1* genes were significantly induced, especially from 8 to 10 dpi, consistent with other stresses. Overall, our results demonstrate that *GmEDS1* family genes play extremely important and varied roles in plant defense responses as they respond rapidly to both abiotic and biotic stresses, thus enhancing the plant’s adaptability to adversity.

## 4 Discussion

### 4.1 Characteristics and phylogenetic relationships of *GmEDS1* gene family members

Although *GmEDS1* has been reported previously, only two *GmEDS1* and one *GmPAD4* were identified in the soybean genome (Wang et al., 2014). In this study, 11 *GmEDS1* family genes were identified and categorized into one of three subfamilies: *EDS1*, *PAD4*, and *SAG101* (**Figures 1**, **4A**). In soybeans, the number of genes in each of these three subfamilies is similar (4, 4, and 3, respectively). Notably, there were no SAG101 orthologs in the monocots rice, maize, and sorghum, consistent with previous reports that *AtSAG101* orthologs were absent in monocots (Wagner et al., 2013; Lapin et al., 2019). The physicochemical properties of GmEDS1 proteins are similar (**Supplementary Table S2**), containing almost the same domains and similar motifs. In Arabidopsis, *Nicotiana benthamiana*, and tomato, the heterodimer cavity formed by the EP domain is essential for TIR-NLR–mediated host cell death, transcriptional reprogramming, and pathogen resistance (Bhandari et al., 2019; Gantner et al., 2019; Lapin et al., 2019). The members of the GmEDS1 family all had EP and lipase 3 domains, indicating that GmEDS1 family proteins may exert similar functions.

A total of 11 *GmEDS1* genes were identified in soybean, nearly three times the number in Arabidopsis. This phenomenon may be explained by whole-genome duplication (WGD) events that have occurred during the evolutionary history of soybean. Soybean has experienced at least two WGD events, approximately 59 million and 13 million years ago, resulting in highly replicated genomes and an increase in the number of many genes compared to the genomes of other diploid species (Schmutz et al., 2010). Duplication events, including segmental duplication and polyploidy events, can generate new genes, which may lead to the production of new functions or functional redundancy in genes, or they may be non-functional pseudogenes (Barker et al., 2012; Lallemand et al., 2020). In our work, intraspecific collinearity analysis showed that there were 7 pairs of fragment duplication events in *GmEDS1* family genes in the soybean genome (**Figure 3A**). Among them, 6 pairs represent the *GmPAD4* subfamily, indicating that the expansion of the *GmPAD4* subfamily may have depended on fragment duplication events.

Another interesting phenomenon was the repetition of two or three adjacent related genes making up a gene cluster; we discovered two events in soybean, *GmEDS1b*/*GmEDS1c*/*GmEDS1d* and *GmSAG101a*/*GmSAG101c*, which were located on Chr6 and Chr13, respectively (**Figure 2**). A total of five genes were involved, including members of the *GmEDS1* and *GmSAG101* subfamilies. We also found the same phenomenon in other species, in Arabidopsis *AtEDS1-80*/*AtEDS1-90*, and the SAG101 subfamily of alfalfa, *Medtr3g467350*/*Medrtr3g467360* and *Medtr6g007975*/*Medtr6g007985*, they were located adjacent to each other on the chromosome (**Figure 1**). The identical chromosomal regions smaller than 200 kb in two or more genes are considered to be tandem duplication events, with the genes performing critical roles in the continued generation of new functions in gene families (Holub, 2001). In addition, plant defense-related genes often appear in clusters (Papadopoulou et al., 1999; Wilderman et al., 2004; Shimura et al., 2007; van Wersch and Li, 2019). We observed the same phenomenon here, and the aggregation of these genes may lead to co expression, giving plants greater ability to simultaneously recognize pathogens and trigger immune responses. However, for the *GmEDS1b*/*GmEDS1c*/*GmEDS1d* and *GmSAG101a*/*GmSAG101c* clusters, we cannot determine whether the genes are closely linked and functional or whether they are non-functional pseudogenes, and this deserves further study.

### 4.2 Upregulation of *GmEDS1* expression in response to abiotic and biotic stress

The binding of transcription factors to *cis*-elements plays a crucial role in the crosstalk between abiotic and biotic stresses in plants (Liu et al., 2024). Our promoter analysis showed that most of the *GmEDS1* gene promoters contained multiple *cis*-elements related to light and stress response, indicating that *GmEDS1*s can be induced by light signals and stress signals to initiate defense responses. Our results indeed showed that the expression levels of *GmEDS1*s were induced under LD conditions (**Figures 7A–D**) and under stress conditions, including salt, drought, CCW infestation, and infection by two SMV strains, SC3 and SC7 (**Figure 8**).

Among the *cis*-elements, the G-box, ABRE, and antioxidant response element (ARE) were relatively dominant; these mainly play roles in light responses, hormone responses, and stress responses. The G-box, with 5′-ACGTGGC-3′ as the core sequence, regulates expression in response to light for many plant genes (Block et al., 1990). In soybean, MYC-related transcriptional activator 2 (MYC2) encodes a bHLH leucine zipper DNA-binding domain, the main regulator of ABA and JA signals. It can specifically recognize the G-box (5′-CACGTG-3′) motif, and plays an important role in *Rsv3*-mediated defense signal transduction in response to SMV (DeMers et al., 2020). This indicates that the light-responsive G-box does far more than just play a role in the light response.

ABA plays a crucial role not only in various stages of plant growth and development, but also in response to abiotic stresses such as drought and salt stress (Huang et al., 2012). The promoter contains conserved ABRE elements, a characteristic of most ABA-responsive genes (Hattori et al., 2002). The transcription factor AREB (ABA Responsive Element-Binding protein) can bind to the ABRE elements and induce gene transcription in response to drought stress; overexpression of *AtAREB1* in soybean enhances its drought resistance (Marinho et al., 2016).

In addition, we found that there were low-temperature response (LTR) elements in the promoters of *GmEDS1a*, *GmEDS1c*, and *GmPAD4d* (**Figure 5**; **Supplementary Table S5**), indicating that these genes may play a role in soybean low-temperature response. Notably, this finding aligns with previous observations in Arabidopsis, after cold exposure, the expression levels of *AtEDS1*, *AtPAD4*, and *AtSAG101* upregulated in roots, meanwhile, AtEDS1, AtPAD4, and AtSAG101 can form a complex and play a role in the freezing signal response. The freezing resistance of EDS1-PAD4-SAG101 functional loss complex is enhanced, which is mediated by ROS and SA (Chen et al., 2015).

In conclusion, owing to the diversity of *cis*-elements in *GmEDS1* promoters, this family of genes has a great functional diversity during plant growth and development, and the pleiotropy of *GmEDS1* genes provides new options for soybean breeding.

### 4.3 The relationship between plant defense and light signaling

Light is an important environmental signal factor, and therefore light quality, light intensity, and photoperiod all affect and regulate many physiological processes of plants. Also, light is necessary for the comprehensive defense response of plants. For instance, the immunity and resistance of Arabidopsis to *Pseudomonas syringae* pv. *tomato* (*Pto*) DC3000 are strongly influenced by the duration of light, with susceptibility to *Pto* DC3000 increasing with shortening day length. Moreover, the expression levels of the key defense genes *PR1*, *PBS3*, and *PAD4* also significantly increase with increased light exposure time (Gangappa and Kumar, 2018). We found many photoresponsive elements in the promoters of *GmEDS1*s, and the expression level of *GmEDS1*s was higher under LD conditions than under SD conditions (**Figures 7A–D**). This may lead to a increase in plant sensitivity to stresses under SD conditions, making plants more susceptible to biotic and abiotic stresses. In our future studies, more experiments and photoperiod regulated mutants are needed to elucidate the molecular mechanism of *GmEDS1s* in the interaction between photoperiod and disease resistance.

The duration of light exposure can also affect defense responses by influencing the content of defense-related substances. Cabbage loopers (*Trichoplusia ni*) mainly feed on Arabidopsis during the day, and the accumulation of JA in Arabidopsis reaches its peak during the day, which is consistent with *T. ni*’s feeding time. The pattern of accumulation of SA is opposite that of JA, manifested as a large accumulation of SA at night, which may enhance the ability of Arabidopsis plants to fight pathogens in the early morning (Goodspeed et al., 2012). Compared with plants grown under SD conditions, plants grown under LD conditions do not show increased accumulation of JA, but instead increase the expression of JA-dependent defense genes, which enhances their defense capabilities (Cagnola et al., 2018). The Arabidopsis resistance gene *HRT* (*HYPERSENSITIVE REACTION TO TURNIP CRINKLE VIRUS*) activates the hypersensitive response and increases resistance to turnip crinkle virus (TCV) through a pathway dependent on both light and SA (Chandra-Shekara et al., 2006). Moreover, SA cannot induce the expression of *EDS1* and *PAD4* in the dark, indicating that SA-mediated expression of *EDS1* and *PAD4* also requires light (Chandra-Shekara et al., 2006). Because soybean is a photoperiod-sensitive crop, further investigation of the crosstalk regulated by *GmEDS1* between plant defense, phytohormones, and photoperiod is of great concern and can provide strategies for the breeding of resistant soybean varieties suitable for different latitudes.

## 5 Conclusion

In summary, we used bioinformatics methods to analyze the phylogeny, evolution, chromosome localization, gene structure, and promoter *cis*-elements of the *GmEDS1* gene family in soybean. A total of 11 *GmEDS1* genes in three phylogenetic subfamilies (EDS1, PAD4, and SAG101) were identified. These genes were unevenly distributed on six different chromosomes, and *GmEDS1b*, *GmEDS1c*, and *GmEDS1d* were clustered together, as were *GmSAG101a* and *GmSAG101c*. All GmEDS1 family proteins have conserved lipase-like and EP domains in the N-terminal and C-terminal regions, respectively. Collinearity analysis indicated that there has been purification selection between the *GmEDS1* and *GmPAD4* subfamily genes during the evolution process, ensuring the conservation of *GmEDS1* and *GmPAD4*. *GmEDS1* promoters are rich in light-responsive, hormone-responsive, and stress-responsive *cis* elements. Prediction of protein–protein interactions indicated that GmEDS1 can interact with defense-related proteins. The *GmEDS1* genes were expressed in all tissues, and the expression level of *GmEDS1*s was much higher under LD conditions than under SD conditions, indicating a relationship between *GmEDS1*s and photoperiod. All of the *GmEDS1*s were upregulated in response to salt, drought, CCW, and SMV treatment, indicating that they play important roles in stress responses and can enhance soybean resistance to both abiotic and biotic stresses. The results not only lay the foundation for further exploring the molecular mechanisms of the *GmEDS1* family members, but also are very useful to soybean breeders.

## Data Availability

The original contributions presented in the study are included in the article/**Supplementary Material**. Further inquiries can be directed to the corresponding authors.

## Supplementary material

Supplementary Figure 1 Comparison of protein sequence similarity between soybean and Arabidopsis EDS1 subfamily. The red lines indicate the lipase 3 domain and the EDS1 EP domain, and the red boxes indicate G-X-S-X-G and S-D-H. The color of amino acids indicates the level of sequence conservation, black: 100% conserved, magenta: 100%-80% conserved, cyan: 80%-60% conserved.

Supplementary Figure 2 Comparison of protein sequence similarity between soybean and Arabidopsis PAD4 subfamily. The red lines indicate the lipase 3 domain and the EDS1 EP domain, and the red boxes indicate G-X-S-X-G and S-D-H. The color of amino acids indicates the level of sequence conservation, black: 100% conserved, magenta: 100%-80% conserved, cyan: 80%-60% conserved.

Supplementary Figure 3 Comparison of protein sequence similarity between soybean and Arabidopsis SAG101 subfamily. The red lines indicate the lipase 3 domain and the EDS1 EP domain, and the red boxes indicate G-X-S-X-G and S-D-H. The color of amino acids indicates the level of sequence conservation, black: 100% conserved, magenta: 100%-80% conserved, cyan: 80%-60% conserved.

Supplementary Figure 4 Motif sequences identified in GmEDS1 family members. The illustrations show the most likely amino acid residue at particular positions throughout the motif, with the size of the letter representing its prevalence.
