# Supplementary figures and images for: Genome-wide identification of GmEDS1 gene family members in soybean and expression analysis in response to biotic and abiotic stresses
Source: Front Plant Sci. 2025 Apr 29;16:1554399. doi: 10.3389/fpls.2025.1554399 (PMC12069366; doi:10.3389/fpls.2025.1554399)

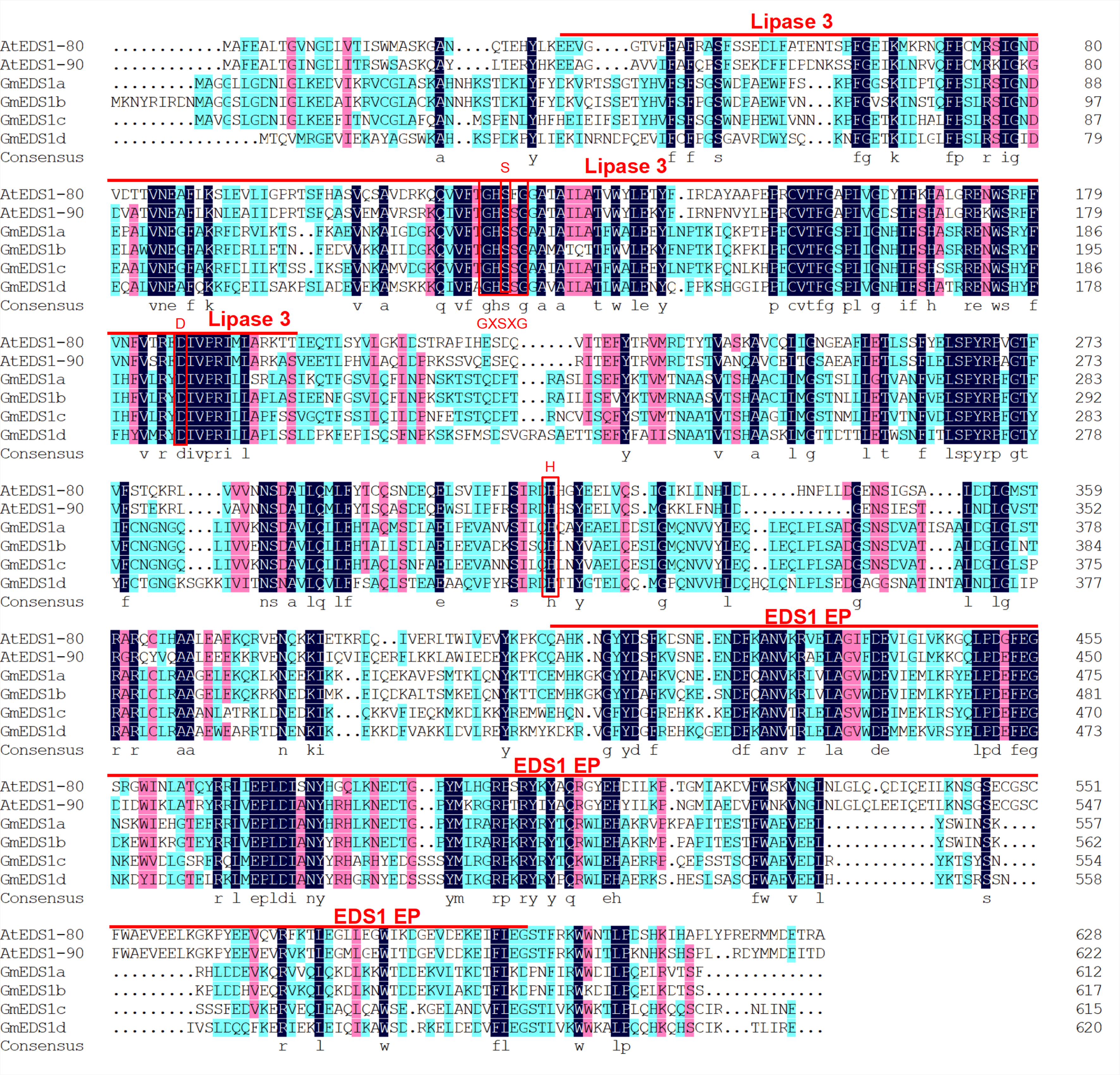

Supplement: Supplementary Figure 1 — Comparison of protein sequence similarity between soybean and Arabidopsis EDS1 subfamily. The red lines indicate the lipase 3 domain and the EDS1 EP domain, and the red boxes indicate G-X-S-X-G and S-D-H. The color of amino acids indicates the level of sequence conservation, black: 100% conserved, magenta: 100%-80% conserved, cyan: 80%-60% conserved. [file Image1.tiff]

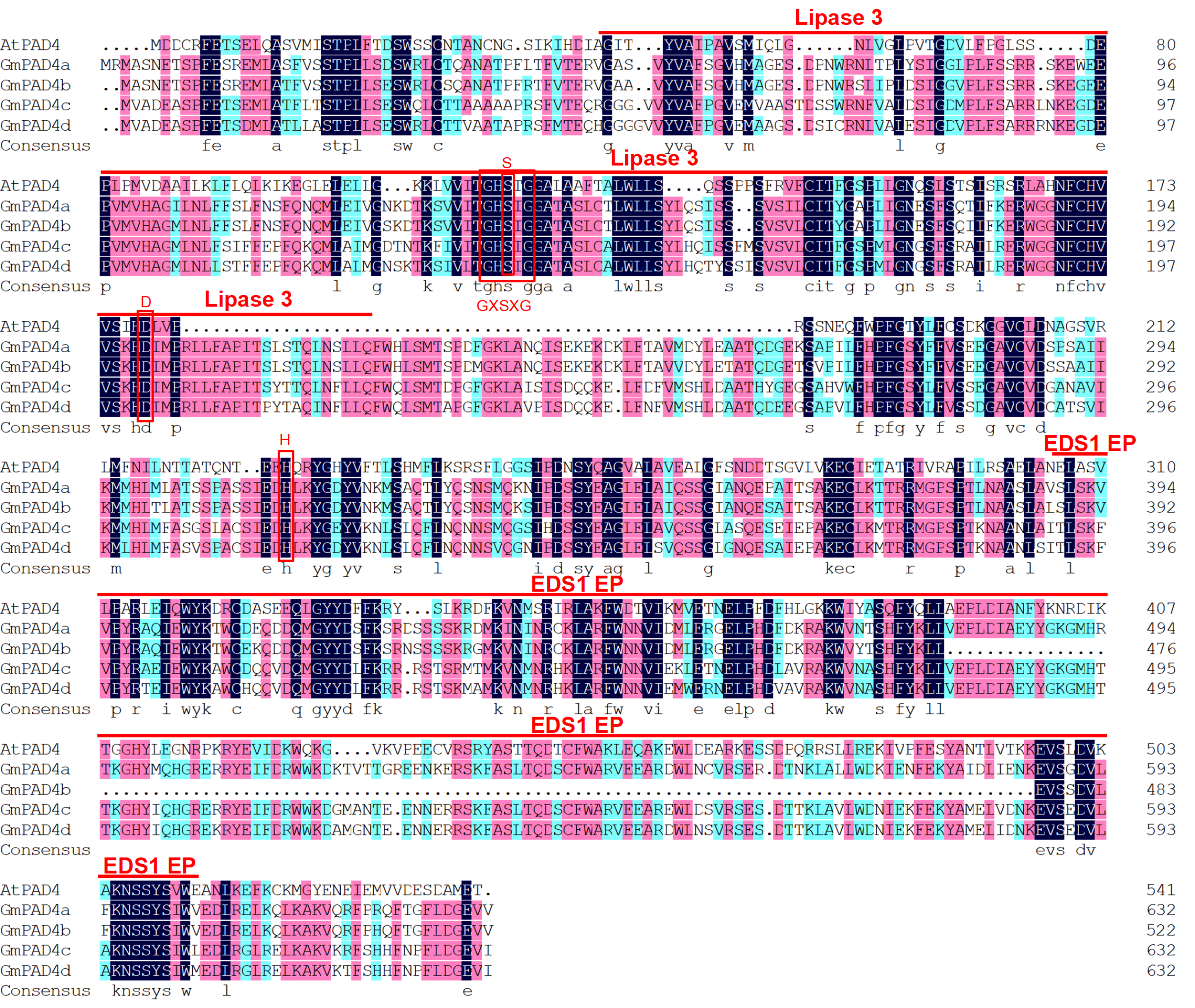

Supplement: Supplementary Figure 2 — Comparison of protein sequence similarity between soybean and Arabidopsis PAD4 subfamily. The red lines indicate the lipase 3 domain and the EDS1 EP domain, and the red boxes indicate G-X-S-X-G and S-D-H. The color of amino acids indicates the level of sequence conservation, black: 100% conserved, magenta: 100%-80% conserved, cyan: 80%-60% conserved. [file Image2.tiff]

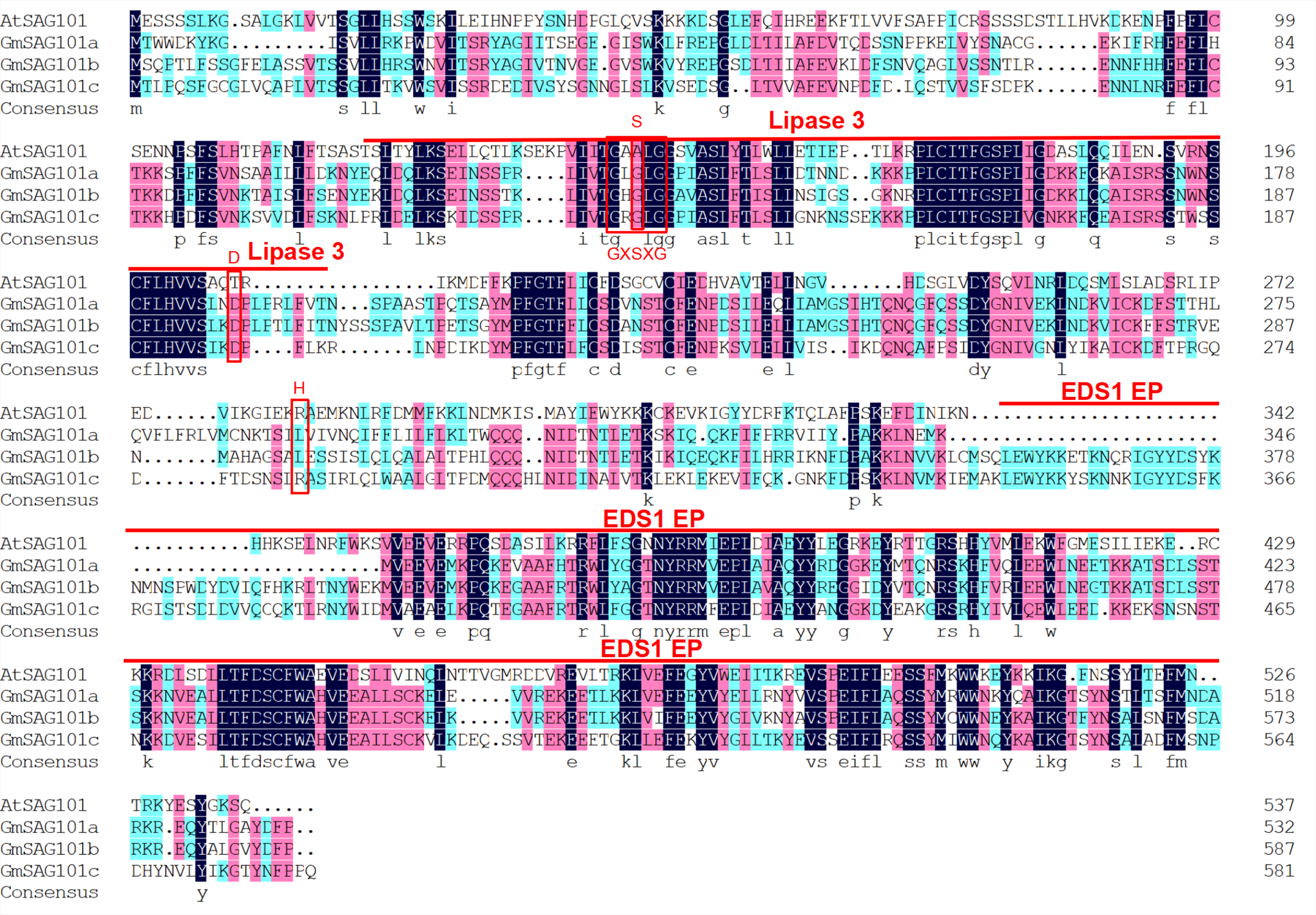

Supplement: Supplementary Figure 3 — Comparison of protein sequence similarity between soybean and Arabidopsis SAG101 subfamily. The red lines indicate the lipase 3 domain and the EDS1 EP domain, and the red boxes indicate G-X-S-X-G and S-D-H. The color of amino acids indicates the level of sequence conservation, black: 100% conserved, magenta: 100%-80% conserved, cyan: 80%-60% conserved. [file Image3.tiff]

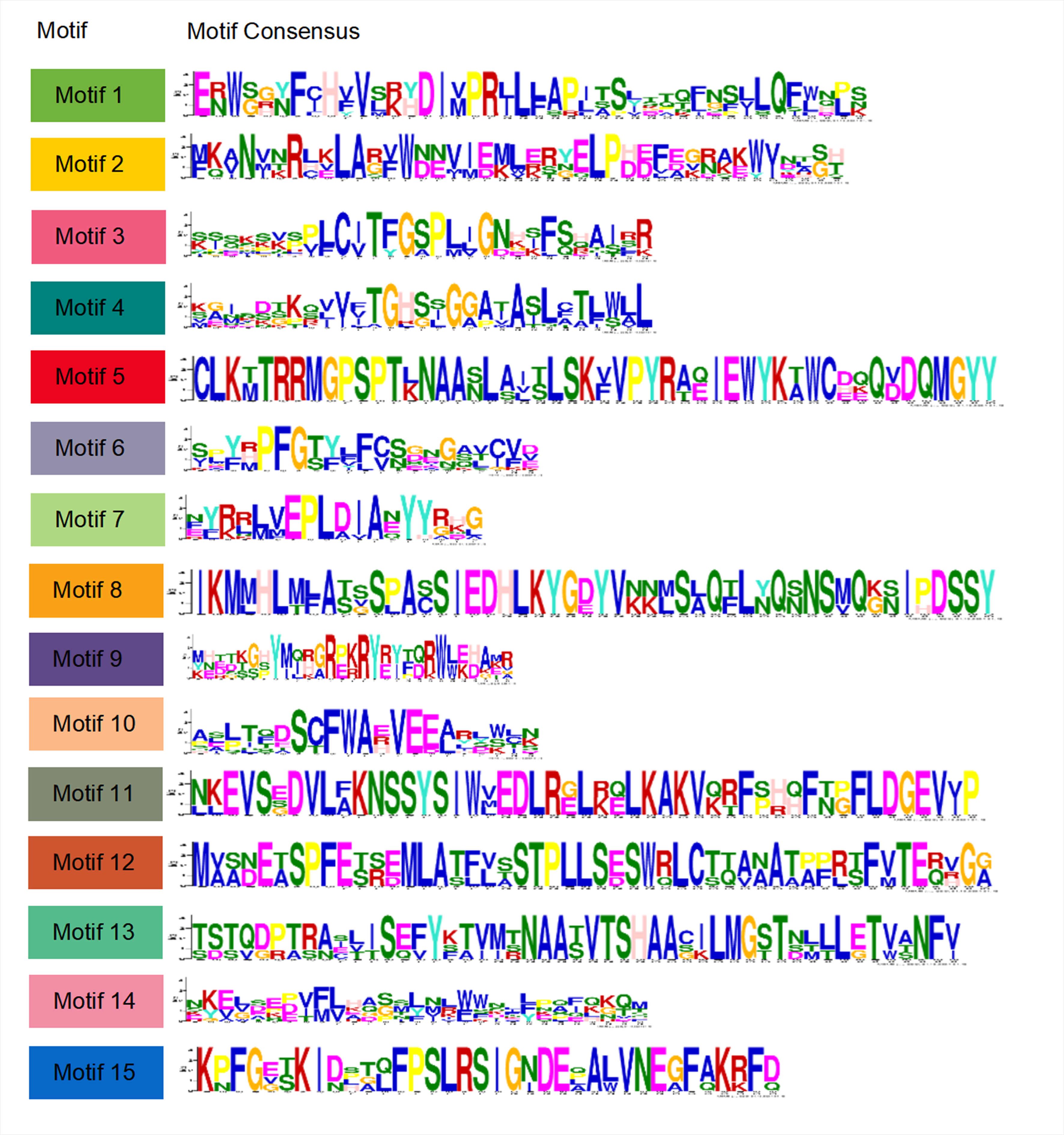

Supplement: Supplementary Figure 4 — Motif sequences identified in GmEDS1 family members. The illustrations show the most likely amino acid residue at particular positions throughout the motif, with the size of the letter representing its prevalence. [file Image4.tiff]
